# Only one cluster per condition is an invalid design for a cluster randomized trial and should be re-labeled a quasi-experimental study. Response to: “Effect of behavioral activation on time and frequency domain heart rate variability in older adults with subthreshold depression: a cluster randomized controlled trial in Thailand”

**DOI:** 10.1186/s12888-022-04491-0

**Published:** 2023-01-13

**Authors:** Yasaman Jamshidi-Naeini, Lilian Golzarri-Arroyo, Abu Bakkar Siddique, Colby J. Vorland, David B. Allison

**Affiliations:** Department of Epidemiology and Biostatistics, Indiana University School of Public Health- Bloomington, 1025 E 7th St, PH 111, Bloomington, IN 47405 USA

**Keywords:** Cluster randomized trial, Degrees of freedom, Inferential testing, Nesting

## Abstract

Ayudhaya et al. examined the effect of Behavioral Activation on daily step count and heart rate variability among older adults with depression in a study labeled a cluster randomized controlled trial (cRCT). However, only one cluster was assigned to either of the study conditions. Such a design would have zero degrees of freedom for inferential testing, because the variation due to cluster membership cannot be estimated apart from the variation due to treatment assignment. Thus, the intervention effect is completely confounded with the cluster effect. The study should be labeled a quasi-experimental study, not a cRCT. Accordingly, the numerical results should be interpreted as associations but not evidence for causal relationships.

Ayudhaya et al. [1] conducted a study to examine the effect of Behavioral Activation (BA) on daily step count (as a physical activity indicator) and heart rate variability (as a depression-related autonomic function indicator) among community-dwelling older adults with mild to moderate depression. The article is labeled as a cluster randomized controlled trial (cRCT). However, there is only one cluster (i.e., hospitals) assigned to either of the study conditions (BA plus usual care or usual care only), which makes the study design invalid for estimating the causal effect of treatment assignment. Forty-one participants were recruited in each hospital after they were assigned to study conditions.

In cRCTs, there can be and typically are correlated data within each cluster (i.e., unit of randomization) so “errors” (loosely speaking, model residuals) cannot be assumed to be independent across study participants within clusters. Moreover, when clusters (rather than individuals) are assigned to study conditions, the study has a hierarchical structure (individuals nested in clusters) [2]. Thus, the degrees of freedom for testing the intervention effect depends both on the number of individual participants and on the number of clusters per condition [3]. A purported cRCT with two conditions and only one cluster assigned to each condition, such as Ayudhaya et al.’s study [1], would have zero degrees of freedom (n1 + n2–2) for inferential testing. That is, the variation due to cluster membership cannot be estimated apart from the variation due to treatment assignment, and a valid analysis of the intervention *effect* could not exist [4].

We commend Ayudhaya et al. for addressing an important question about depression among the older population, and for their collegiality in providing clarification about their study design. The authors’ clarification confirmed our understanding from reading the published methods.

Because in Ayudhaya et al.’s study [1] the intervention effect is completely confounded with the cluster effect, it should be labeled a quasi-experimental study, not a cRCT. Accordingly, the numerical results should be interpreted as associations between BA and the outcomes but not evidence for causal relationships [5].

## Data Availability

Not applicable.

## Abbreviations

BA: Behavioral Activation
cRCT: Cluster Randomized Controlled Trial
